# A functional polymorphism in the paired basic amino acid-cleaving enzyme 4 gene confers osteoarthritis risk in a population of Eastern China

**DOI:** 10.1590/1678-4685-GMB-2019-0115

**Published:** 2020-02-10

**Authors:** Jin He, Haoyu Yang, Zhonghua Xu, Jin Li, Gang Chen, Lifeng Jiang, Lidong Wu, Xindie Zhou

**Affiliations:** 1 Department of Orthopedics, Jintan Hospital Affiliated to Jiangsu University, Changzhou, China.; 2 Department of Orthopedics, Wuxi No.9 People’s Hospital Affiliated to Soochow University, Wuxi, Jiangsu, China.; 3 Department of Orthopedic Surgery, the Second Affiliated Hospital of Jiaxing University, Jiaxing, China.; 4 Department of Orthopedic Surgery, The Second Affiliated Hospital, Zhejiang University School of Medicine, Hangzhou, China.; 5 Department of Orthopedics, The Affiliated Changzhou No.2 People’s Hospital of Nanjing Medical University, Changzhou, China.

**Keywords:** ACE4, osteoarthritis, bioinformatics analysis, single-nucleotide polymorphism

## Abstract

Paired basic amino acid-cleaving enzyme 4 (PACE4), a proprotein convertase, is involved in the activation of aggrecanases (ADAMTS-4 and ADAMTS-5) in osteoarthritic and cytokine-stimulated cartilage. Activated aggrecanases cause aggrecan degradation and thus, contribute to osteoarthritis (OA). In this study, we investigated the association between *PACE4* gene polymorphisms and OA risk. One single-nucleotide polymorphism (rs4965833) in the *PACE4* gene was genotyped in 432 OA patients and 523 healthy controls using matrix-assisted laser desorption/ionization time-of-flight mass spectrometry. Quantitative reverse transcription PCR (qRT-PCR) was used to determine the relative expression of *PACE4* in blood samples from 90 OA patients (30 for each genotype). The relative expression level of *PACE4* mRNA was higher in the GG genotype as compared to the AA/AG group. Moreover, the *PACE4* rs4965833 polymorphism was associated with increased risk of OA, especially among individuals aged ≥55 years and with a body mass index ≥25. There was no significant association between the *PACE4* rs4965833 polymorphism and clinical parameters of OA patients, such as erythrocyte sedimentation rate, C-reactive protein, Visual Analog Scale for pain and Lequesne’s index. In conclusion, the rs4965833 polymorphism in the 3’-UTR of *PACE4* is associated with OA susceptibility.

## Introduction

Osteoarthritis (OA) is the most common form of arthritis among the elderly and one of the leading musculoskeletal causes of disability in Western countries (Dahaghin *et al.*, 2005). OA limits movement, particularly walking, and affects participation in everyday activities and quality of life (Palazzo *et al.*, 2016). OA in the hips and knees is associated with the greatest burden in affected inviduals since pain and stiffness in these large, weight-bearing joints often lead to significant disability (Litwic *et al.*, 2013). OA is associated with obesity and other cardiovascular risk factors, such as diabetes, dyslipidemia, hypertension, and insulin resistance (Velasquez and Katz, 2010). Moreover, genetic variability influences the pathogenesis of OA (Norcini and Shea, 1990).

Active A disintegrin and metalloproteinase with thrombospondin motif (ADAMTS)-4 and ADAMTS-5 are responsible for proteolytic degradation of the major cartilage macromolecules, aggrecan and type II collagen, which is a key pathological event in OA (Little and Fosang, 2010). Paired basic amino acid-cleaving enzyme 4 (PACE4) is a proprotein convertase responsible for activation of aggrecanases in osteoarthritic and cytokine-stimulated cartilages (Malfait *et al.*, 2012). Previous reports show that *PACE4* mRNA levels increase markedly during chondrogenic differentiation and knockdown of *PACE4* expression significantly reduces chondrogenic differentiation (Yuasa *et al.*, 2012). *PACE4* knockout significantly protects mice from OA pain (Malfait *et al.*, 2012). Thus, these observations suggest that overexpression of *PACE4* (activator of ADAMTS-4/5) promotes the expression or release of proteoglycanases, which contribute to the development of OA. Furthermore, the increased levels of PACE4 also intensify the pain response.

MicroRNAs (miRNAs) are endogenous non-coding RNAs that bind to their complementary sites on the 3’-UTR of the target mRNAs to mediate mRNA degradation and repression of translation (Chuang and Jones, 2007; Sato *et al.*, 2011). Genetic variations in the 3’-UTR may affect the binding of miRNA to its target mRNA and thereby, confer susceptibility to conditions such as OA. Here, a case-control study was conducted to investigate the role of polymorphisms in the 3’-UTR of PACE4 in the regulation of PACE4 expression via miRNAs and evaluate whether the polymorphism confers susceptibility to OA.

## Materials and Methods

### Study subjects

A total of 432 OA patients and 523 healthy controls were recruited from Jintan Hospital Affiliated to Jiangsu University (Changzhou, China), the Second Affiliated Hospital of Jiaxing University (Jiaxing, China) and the Second Affiliated Hospital, Zhejiang University School of Medicine (Hangzhou, China). All participants were genetically unrelated ethnic Han Chinese. Radiographic confirmation of the diagnosis of each patient was performed using the Kellgren–Lawrence grade (K–L grade) system. Inclusion criteria were: (1) symptoms and/or signs of OA; (2) radiographic abnormalities (K–L grade ≥2); and (3) no evidence of any other form of arthritis. The functional or symptomatic status of patients was assessed using Lequesne’s functional index. Pain was evaluated using the Visual Analog Scale (VAS). Controls were selected from patients attending the general surgery and orthopedics clinics of the three hospitals at the time of sample collection. Individuals with any systemic inflammatory or autoimmune disorder or any type of malignant or chronic illness were excluded. A questionnaire was designed to collect general information (e.g. age, sex, body mass index [BMI]) and biochemical data (e.g. erythrocyte sedimentation rate [ESR] and C-reactive protein [CRP]) of OA from cases and controls.

This study was approved by the Ethics Committees of the Jintan Hospital Affiliated to Jiangsu University (ID: 20190005), the Second Affiliated Hospital of Jiaxing University (ID: jxey-2020SZ2044) and the Second Affiliated Hospital, Zhejiang University School of Medicine (ID: Pre-review study 2016-088). All patients provided written informed consent prior to their participation.

### Single-nucleotide polymorphism (SNP) selection and genotyping

The SNPs in the 3’-UTR of *PACE4* gene were selected for further investigation by dbSNP (https://www.ncbi.nlm.nih.gov/projects/SNP/) using the following parameters: (1) variation: SNP; (2) function: 3’-UTR; (3) global minor allele frequency (MAF): 0.05–0.5; (4) validation status: by 1,000 Genomes. The miRNAs potentially targeting the 3’-UTR of *PACE4* were predicted by MirSNP (http://bioinfo.bjmu.edu.cn/mirsnp/search/). In brief, 2 mL of peripheral blood was collected from each subject, transferred to a test tube containing ethylenediaminetetraacetic acid (EDTA) and stored at -80 °C prior to use. DNA was extracted from the blood examples using a QIAamp DNA blood mini-kit (Qiagen, Hilden, Germany). The concentration and purity of the extracted DNA were estimated by measuring the optical density (OD) at wavelengths of 260 and 280 nm. The extracted DNA with a concentration of 50 μg/mL or OD_260_/OD_280_ = 1.8–2.0 was used for genotyping. Genotyping was conducted by matrix-assisted laser desorption/ionization time-of-flight mass spectrometry (MALDI-TOFMS) using a MassARRAY system (Sequenom, San Diego, CA, USA). Completed genotyping reactions were spotted onto a 384-well spectroCHIP system (Sequenom) using a MassARRAY nano-dispenser (Sequenom) and analyzed by MALDI-TOFMS. Genotypes were called in real time on MassARRAY RT 3.1 and analyzed on MassARRAY Typer 4.0 (both Sequenom). Approximately 5% of the samples were randomly selected for a blinded retest.

### Quantitative reverse transcription-polymerase chain reaction (qRT-PCR)

Total RNA was isolated from whole blood samples obtained from 90 OA patients (30 patients for each genotype) using the TRIzol reagent (Invitrogen, Carlsbad, CA, USA) according to the manufacturer’s instructions. *PACE4* expression was detected by qRT-PCR using SYBR Green I chemistry. Forward and reverse primers used for PCR were as follows: 5’-CTATGGATTTGGTTTGGTGGAC-3’, 5’-AGGCTCCATTCTTTCAACTTCC-3’ (PACE4); 5’-CTGCACCACCAACTGCT TAG-3’, 5’-AGGTCCACC ACTGACACGTT-3’ (GAPDH). Gene expression levels were normalized to that of GAPDH and fold changes in expression were calculated using the 2^-DDCT^ method.

### Statistical analysis

All data were expressed as mean ± standard deviation (SD). Genotype distributions in the controls were tested to confirm Hardy–Weinberg equilibrium (HWE) using the χ^2^ test. Qualitative data were compared using the χ^2^ test. Differences in continuous variables between cases and controls were analyzed using Student’s *t*-test. Groups were compared by one-way analysis of variance (ANOVA). Association between PACE4 rs4965833 polymorphism and OA risk was evaluated through logistic regression analyses and using odds ratios (ORs) and 95% confidence intervals (CIs). All statistical analyses were performed using SAS 9.1.3 (SAS Institute, Cary, NC, USA). *P* < 0.05 was considered to indicate statistical significance.

## Results

### Characteristics of the study population

Demographic variables and baseline characteristics of the participants are shown in Table 1. There were no significant differences between OA patients and controls in terms of sex, age or BMI. Affected leg, ESR, CRP, VAS, Lequesne’s index, and K–L grade are listed in the left column and 44.9% of participants were classified as grade 2.

**Table 1:** Subjects demographics and risk factors in knee osteoarthritis.

Variables	Patients (n=432)	Controls (n=523)	*P*
Sex			0.555
Male	190 (44.0%)	240 (45.9%)	
Female	242 (56.0%)	283 (54.1%)	
Age (years)	62.32 ± 7.98	62.21 ± 7.87	0.829
BMI (Kg/m^2^)	25.53 ± 2.67	25.38 ± 2.80	0.410
Affected leg			
Left	287 (66.4%)	-	
Right	145 (33.6%)	-	
ESR (mm/h)	17.47 ± 14.84	-	
CRP (mg/L)	18.44 ± 17.22	-	
VAS	6.51 ± 1.37	-	
Lequesnes’ index	14.75 ± 1.78	-	
kellgren-Lawrence grade			
II	194 (44.9%)	-	
III	155 (41.7%)	-	
IV	23 (6.2%)	-	

BMI, Body Mass Index; ESR, erythrocyte sedimentation rate; CRP, C-Reactive protein; VAS, visual Analogue Scale.

### *PACE4* rs4965833 polymorphism alteration of miR-7 binding

DbSNP database analysis identified 12 SNPs according to our selection criteria. Of these, four SNPs were associated with miRNA binding (Table 2). Sequencing results showed only one SNP (rs4965833) was positive. The miRNAs hsa-miR-7 and hsa-miR-4432 were predicted to target the *PACE4* rs4965833 polymorphism. Since few studies have focused on hsa-miR-4432, we chose hsa-miR-7 for further research. Hsa-miR-7 could not target the locus of rs4965833 polymorphism if nucleotide A was changed into G.

**Table 2:** SNPs located in the PACE4 gene 3’-UTR and the predicted miRNAs.

SNP	Chromosome	HGVS Names	miRNA
Rs2949	15:101347562	NM_138320.1:c.1859-15531G>A	miR-5589-3P
			miR-586
Rs1030	15:101347589	NM_138319.3:c.1859-15558A>T	miR-452-5P
			miR-4676-3P
Rs273595	15:101304719	NM_001291309.1:c.*539G>A	miR-3545-5P
			miR-483-3P
			miR-539-3P
Rs4965833	15:101364879	NM_002570.4:c.1858+1317C>A	miR-4432
			miR-7-5P

### Association between *PACE4* rs4965833 polymorphism and OA risk

Table 3 shows the genotype and allele distributions for the *PACE4* rs4965833 polymorphism in the OA cases and the controls. No significant deviation from HWE was found for this SNP in the controls (*P* > 0.05). The GG genotype significantly affected the increased risk of OA compared with the A genotype. Furthermore, the GG+AG genotype also significantly increased the risk of OA. The significant association also held true after adjustment for sex and age. The G allele of the rs4965833 polymorphism was significantly correlated with increased risk of OA.

**Table 3:** Logistic regression analysis of associations between rs4965833 polymorphism and risk of knee osteoarthritis.

Genotype	Cases^a^(n=432)		Controls^a^(n=523)		OR (95% CI); *P*	Adjusted OR (95% CI)^b^; *P*
	n	%	n	%		
AG vs. AA	185/175	42.8/40.5	209/245	40.0/46.8	1.24(0.94,1.64); 0.129	1.24(0.94,1.64); 0.123
GG vs. AA	70/175	16.2/40.5	66/245	12.6/46.8	**1.49(1.01,2.19); 0.046**	**1.49(1.01,2.20); 0.043**
GG+AG vs. AA	255/175	59/40.5	275/245	52.6/46.8	**1.30(1.00,1.68); 0.048**	**1.30(1.01,1.69); 0.045**
GG vs. AG+AA	70/360	16.2/83.3	66/454	12.6/86.8	1.34(0.93,1.92); 0.117	1.34(0.93,1.93); 0.113
G vs. A	325/535	37.6/61.9	341/699	32.6/66.8	**1.24(1.03,1.50); 0.023**	

a The genotyping was successful in 430 cases and 520 controls.

b Adjusted for sex and age.

Bold values are statistically significant (*P* < 0.05).

Subgroup analyses were conducted according to sex, age and BMI (Table 4). Results showed the *PACE4* rs4965833 polymorphism was associated with the increased risk of OA among old people (≥55 years) under the homozygous and dominant models. Subgroup analysis according to BMI further indicated this significant association in the BMI ≥25 kg/m^2^ subgroup. Then other clinical parameters of OA (e.g. ESR, CRP, VAS and Lequesne’s’ index) were compared among different genotypes (Table 5). For the rs4965833 polymorphism, no significant effect on OA risk was observed in terms of affected leg, ESR, CRP, VAS, Lequesne’s index or K–L grading.

**Table 4:** Stratified analyses between PACE4 rs4965833 polymorphism and the risk of osteoarthritis.

	Rs4965833(case/control)						
Variable	AA	AG	GG	AG vs. AA	GG vs. AA	AA+AG vs. AA	GG vs. AG+AA
Sex							
Male	74/103	78/105	36/30	1.03(0.68,1.57); 0.876	1.67(0.95,2.95); 0.077	1.18(0.80,1.73); 0.416	1.64(0.97,2.78); 0.066
Female	101/142	107/104	34/36	1.45(1.00,2.10); 0.051	1.33(0.78,2.26); 0.298	1.42(1.00,2.00); 0.049	1.12(0.68,1.85); 0.666
Age (years)							
<55	29/37	28/33	9/12	1.08(0.54,2.18); 0.824	0.96(0.36,2.58); 0.931	1.05(0.55,2.01); 0.886	0.92(0.36,2.34); 0.863
≥55	146/208	157/176	61/54	1.27(0.94,1.72); 0.120	**1.61(1.05,2.46); 0.028**	**1.35(1.02,1.79); 0.036**	1.43(0.96,2.13); 0.076
BMI (kg/m^2^)							
<25	82/117	68/83	29/25	1.17(0.76,1.79); 0.474	1.66(0.90,3.03); 0.101	1.28(0.87,1.90); 0.216	1.56(0.87,2.75); 0.135
≥25	88/133	117/126	47/36	1.40(0.97,2.03); 0.071	**1.97(1.18,3.29); 0.009**	**1.53(1.08,2.16); 0.016**	**1.65(1.03,2.64); 0.036**

Bold values are statistically significant (*P* < 0.05).

**Table 5:** Comparison of studied data according to PACE4genotypes in all OA cases.

		OA(n=432)							
PACE4 rs4965833		AA (n=175)	AG (n=185)	GG (n=70)	*P*	AG+GG (n=255)	*P*	AA+AG (n=360)	*P*
Affected leg	Left/right, n	116/59	123/62	48/22	0.938	171/84	0.867	239/121	0.723
ESR, mm/h	SD ± SEM	17.93 ± 16.60	17.04 ± 13.32	16.86 ± 13.83	0.809	16.99 ± 13.43	0.518	17.48 ± 14.99	0.750
CRP, mg/L	SD ± SEM	18.98 ± 17.52	17.30 ± 14.36	20.35 ± 22.77	0.400	18.14 ± 17.09	0.619	18.12 ± 15.97	0.322
VAS	SD ± SEM	6.53 ± 1.40	6.49 ± 1.42	6.56 ± 1.16	0.932	6.51 ± 1.35	0.873	6.51 ± 1.41	0.798
Lequesnes’ index	SD ± SEM	14.79 ± 1.82	14.64 ± 1.78	14.93 ± 1.69	0.463	14.72 ± 1.76	0.662	14.71 ± 1.80	0.357
KL grading	III+IV/II, n	31/144	41/144	8/62	0.134	206/49	0.694	288/72	0.092

**Figure 1:**
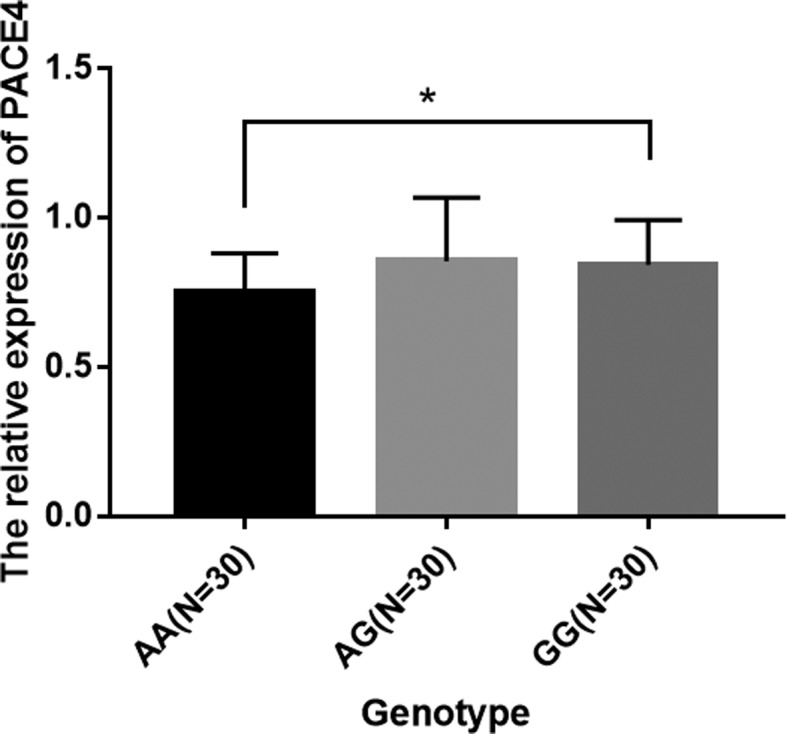
Expression levels of PACE4 in three different genotypes.

RT-PCR analysis showed that *PACE4* expression was significantly elevated in the GG genotype in comparison with that in the AA genotype (*P* < 0.05) (Figure 1).

## Discussion

In this study, we found that the *PACE4* gene rs4965833 polymorphism conferred susceptibility to OA, especially among subjects aged ≥55 years or with BMI ≥25 kg/m^2^. However, there is no evidence that this SNP is associated with the clinical parameters of OA patients.

Many recent studies have investigated the role of PACE4 in the development of cancer (Kang *et al.*, 2014; Lin *et al.*, 2015; Wang *et al.*, 2015; Yao *et al.*, 2015). PACE4 expression was found to be significantly higher in non-small cell lung cancer (NSCLC) tissue than that in normal lung tissue and was associated with worse survival in patients with NSCLC (Lin *et al.*, 2015). Additionally, miR-124 has been shown to inhibit the proliferation and migration of prostate cancer cells via the PACE4 pathway (Kang *et al.*, 2014). However, few studies have elucidated the effect of PACE4 on the pathogenesis of OA. Malfait *et al*. (2012) evaluated the association of 10 *PACE4* gene polymorphisms with the risk of symptomatic knee OA in a Caucasian cohort of 600 OA cases and 432 controls. This study revealed that the *PACE4* rs900414 polymorphism is associated with symptomatic OA compared with asymptomatic OA. This cohort study focused on the effect of the *PACE4* rs900414 polymorphism on OA pain (Malfait *et al.*, 2012); however, in the present study, we calculated the genotype and allele distributions of SNPs to evaluate the association between these SNPs and OA risk. These SNPs may serve as potential biomarkers for early prevention and diagnosis of OA. In our study, bioinformatic analysis indicated that the rs4965833 polymorphism in the 3’-UTR of PACE4 affects its binding to miR-7 such that binding of miR-7 to the mRNA of PACE4 is abolished by substitution of the A nucleotide to G. Proteolytic degradation of the major cartilage macromolecules, aggrecan and type II collagen, is a key pathological event in OA (Malfait *et al.*, 2008). MiR-7 regulates IL-1β-induced extracellular matrix degeneration by targeting growth differentiation factor 5 in human nucleus pulposus cells (Liu *et al.*, 2016). MiR-7 also regulates the expression of matrix metalloproteinase (MMP)-2 and MMP-9 in colon cancer/glioblastoma cells (Wu *et al.*, 2011; Zeng *et al.*, 2016). MiR-7 downregulation induces excessive collagen expression in localized scleroderma (Etoh *et al.*, 2013). Furthermore, activation of ADAMTS-4/5 by PACE4 results in degradation of the cartilage matrix, which in turn promotes the OA development.

To verify our hypothesis, we performed a case-control study to evaluate the effect of the *PACE4* rs4965833 polymorphism on the risk of OA. Our results indicated this polymorphism was associated with an increased risk of OA. Additionally, the mutant GG genotype of the rs4965833 polymorphism is associated with higher levels of *PACE4* mRNA compared to the AA genotype. These results supported our hypothesis on the above assumption. The power analysis indicated that this study had a power of 35.0% to detect the effect of rs4965833 polymorphism on OA susceptibility, assuming an OR of 1.24.

Although positive results were observed, some limitations of this study need to be addressed. First, the results may be affected by confounding factors, such as smoking and drinking habits. Second, the sample size of this study was relatively small, which may make the study underpowered. Third, only one SNP of the *PACE4* gene was genotyped and thus, gene coverage was incomplete. Fourth, selection bias could be not avoided because this was a hospital-based study and may not be representative of the general population. Fifth, luciferase reporter gene experiments should be conducted to confirm that *PACE4* is a target gene of miR-7. Sixth, the positive findings of this study are only for the Han population in East China and cannot be applied to other regions and ethnic groups. Notably, this is the first study exploring the association between the *PACE4* rs4965833 polymorphism and OA risk and hence, may guide further studies in this area.

In conclusion, the *PACE4* rs4965833 polymorphism is a genetic contributor to OA risk in a population of Eastern China. Later, another studies in other regions or ethnic groups are needed to confirm this finding.
